# The Variant at TGFBRAP1 but Not TGFBR2 Is Associated with Antituberculosis Drug-Induced Liver Injury

**DOI:** 10.1155/2019/1685128

**Published:** 2019-08-22

**Authors:** Jingwei Zhang, Zhenzhen Zhao, Hao Bai, Lin Jiao, Qian Wu, Tao Wu, Tangyuheng Liu, Xuejiao Hu, Jiajia Song, Mengyuan Lyv, Binwu Ying

**Affiliations:** Department of Laboratory Medicine, West China Hospital, Sichuan University, Chengdu, Sichuan, China

## Abstract

**Background:**

TGFBRAP1 and TGFBR2 play important roles in the TGF-*β*/smad signalling pathway and may disturb liver homeostasis by regulating liver injury and renewal. However, little is known about the association between their genetic polymorphisms and antituberculosis drug-induced liver injury (ATDILI), so we explored the association between their variants and the susceptibility to ATDILI.

**Materials and Methods:**

A total of 746 tuberculosis patients were prospectively enrolled, and fifteen selected SNPs were genotyped. The allele, genotype, and genetic model frequencies of the variants were compared between patients with or without ATDILI, as well as the joint effect analysis of SNP-SNP interactions. The odds ratio (OR) with the corresponding 95% confidence interval (CI) was calculated.

**Results:**

The A variant at rs17687727 was significantly associated with an increased risk for ATDILI (OR 1.55; 95% CI: 1.08–2.22; *p* = 0.016), which is consistent with the results in the additive and dominant models. Other allele, genotype, and genetic model frequencies were similar in the two groups for the other fourteen SNPs (all *p* > 0.05).

**Conclusion:**

Our study first implied that the A variant of rs17687727 in TGFBRAP1 influenced the susceptibility to ATDILI in first-line antituberculosis combination treatment in the Han Chinese population in a dependent manner.

## 1. Introduction

Tuberculosis (TB) is a leading infectious disease, with approximately 10 million new cases and 1.6 million deaths in 2017 as reported by the WHO [1]. In addition, China had the second largest number of new TB cases in the world in 2017 [1]. At present, although significant progress has been made in the treatment of antituberculosis drugs, the combination of isoniazid, rifampicin, pyrazinamide, and streptomycin is still recommended by the WHO as the standard chemotherapy to cure tuberculosis effectively and prevent the production of resistant bacteria [1]. Although effective, 2.0–28.0% of patients receiving the combination therapy developed antituberculosis drug-induced liver injury (ATDILI). The incidence fluctuates depending on the characteristics of the particular cohort, drug regimens involved, threshold used to define hepatotoxicity, and monitoring and reporting practices [2, 3]. Because of atypical symptoms and nonspecific diagnostic criteria, it is difficult to make an early and accurate diagnosis of ATDILI, which can result in delayed treatment. Whereas mild ATDILI can recover by itself after withdrawing related drugs, severe ATDILI can cause fulminant hepatic failure, liver transplantation, or even death, resulting in a heavy social burden [4]. Prediction of hepatotoxicity is critical in the treatment of TB and can guide the choice of safe medicines.

The pathogenesis of ATDILI mainly involves four mechanisms: drug metabolism, oxidative stress, mitochondrial dysfunction, and immune regulation and inflammatory response [3, 5]. Although the exact mechanisms are not yet fully understood, genetic polymorphisms of genes related to hypothesis have been extensively studied, which helped to clarify the pathogenic mechanisms, and there is growing evidence that genetic vulnerability of related genes may be involved in the pathogenesis [6]. Single nucleotide polymorphisms (SNPs), which are the most common genetic variants, have been shown to have ATDILI clinical guidance value. For example, the associations of “slow acetylation” phenotypes of the NAT gene with increased rates of toxic reactions have been incorporated into the FDA's drug label for isoniazid treatment [7]. In addition, in studies of other genes, such as drug metabolizing enzymes, accumulation of bile acids, lipids, and haem metabolites, immune adaptation, and oxidant challenge, the association still needs further verification [7]. However, these studies also provide novel insight into our better understanding of ATDILI. It is necessary and urgent to clarify the pathogenesis of ATDILI and discover key molecules in the progression as targets for diagnosis and treatment.

Transforming growth factor-beta (TGF-*β*) is a key regulator of liver physiology and pathology, contributing to all stages of disease progression, from initial liver injury through inflammation, wound healing, tissue homeostasis, fibrosis, immune modulation, and hepatocellular carcinoma (HCC) [8]. The TGF-*β*/smad signalling pathway can regulate the function of lymphocytes and macrophages; as a result, inflammatory-related cytokine changes in dose and time-space effects may be involved in liver homeostasis [9]. Therefore, it is reasonable to infer the potential involvement of the TGF-*β*/smad signalling pathway in ATDILI. There are three types of TGF-*β* transmembrane receptors: TGF-*β* receptor 1 (TGFBR1), TGF-*β* receptor 2 (TGFBR2), and TGF-*β* receptor 3 (TGFBR3). Only TGFBR2 can bind TGF-*β*1, and then, it promotes TGFBR1 phosphorylation and recruitment to trigger the formation of a heterotetrameric complex of TGFBR1 and TGFBR2. Then, activated receptor complexes mediate canonical TGF-*β* signalling through phosphorylation of the receptor-associated SMADs (smad2/3). After phosphorylation, smad2/3 forms a trimeric complex with smad4, which translocates to the nucleus and associates with other transcription factors to regulate gene expression. TGF-*β* receptor-associated protein 1 (TGFBRAP1) was recently shown to be the molecular chaperone of smad 4. TGFBRAP1 carries smad 4 to the activated TGFBR2 complex and promotes the phosphorylation of smad 2/3 [10]. The mutant form of TGFBRAP1 may inhibit the signalling pathway through interference complex formation [10]. Therefore, as the important role of TGFBRAP1 and TGFBR2 in the signalling pathway, genetic gene polymorphisms of TGFBRAP1 and TGFBR2 have been researched in hepatocellular carcinomas and hepatitis C infection, which indicated that genetic polymorphisms of TGFBRAP1 and TGFBR2 may disturb the regulation in liver injury and renewal [11–13]. However, to the best of our knowledge, no genetic associations between TGFBRAP1 and TGFBR2 variants and ATDILI have been reported.

Therefore, considering the heavy load of tuberculosis in China, the aim of the present study was to explore the possible association between TGFBRAP1 and TGFBR2 gene polymorphisms with the risk of ATDILI in the Han Chinese population.

## 2. Subjects and Methods

### 2.1. Subjects

Ethical approval for this study was obtained from the Institutional Review Board of the West China Hospital of Sichuan University. We recruited 1060 highly suspicious tuberculosis patients at the West China Hospital between December 2014 and April 2018 consecutively. In total, 817 tuberculosis patients were confirmed by experienced respiratory physicians with a clear tuberculosis diagnosis. All patients underwent standard short-course chemotherapy consisting of isoniazid, rifampicin, pyrazinamide, and ethambutol for six months in accordance with the approved guidelines. Treatments were adjusted accordingly if any patient developed definite ATDILI. The definition of drug-induced liver injury we used was based on the National Institutes of Health and Common Toxicity Criteria for Adverse Events v5.0 (CTCAE v5.0), unless stated otherwise [14]. The inclusion criteria for the ATDILI group were as follows: (a) normal serum alanine aminotransferase (ALT) (0–40 IU/L) and aspartate aminotransferase (AST) (0–40 IU/L) before treatment; (b) ALT and/or AST levels ≥3 × upper limit of normal (ULN) (120 IU/L) with hepatitis symptoms such as jaundice, nausea, vomiting, and abdominal pain; (c) ALT and/or AST levels ≥5 × ULN (200 IU/L), with or without symptoms; (d) total bilirubin (TBIL) ≥1.5 × ULN (42 *μ*mol/L); and (e) no administration of other potentially hepatotoxic drugs two weeks before the occurrence of ATDI (LIDI/> HIV) (no history of HIV treatment [14, 15]). The inclusion criteria for the non-ATDILI group were normal serum ALT, AST, and TBIL before and after treatment. Ultimately, 746 tuberculosis patients receiving first-line treatment were enrolled. The process of study enrolment is shown [Supplementary-material supplementary-material-1] Figure. Demographic and clinical characteristics of the enrolled patients were obtained from electronic medical records.

### 2.2. Sample Genotyping and Data Collection

Genomic DNA was extracted from three millilitres (ml) of EDTA anticoagulated whole blood obtained from all participants for genotyping by the QIAamp® DNA Blood Mini Kit (Qiagen, Germany) according to the manufacturer's instructions. The DNA samples were stored at −80°C until further analysis. The SNP genotyping work was conducted by a custom-by-design 2x48-Plex SNP scan TM Kit (Cat#: G0104, Gene sky Biotechnologies Inc, Shanghai, China), as described previously [16]. Along with treatment, biochemical and haematological analyses were performed twice a month during the first two months and monthly in the subsequent four months. Test results and clinical symptoms were recorded to assess ATDILI.

### 2.3. The Clinical Definition of ATDILI Severity

The severity of hepatotoxicity is classified into three major categories according to the WHO Toxicity Classification Standards: grade 1 (mild) ALT <5 × ULN (200 IU/L), grade 2 (moderate) ALT level higher than 5 × ULN but less than 10 × ULN, and grade 3 (severe) ALT levels ≥10 × ULN (400 IU/L) [17].

### 2.4. Candidate Single Nucleotide Polymorphism Selection

Candidate SNPs were selected by the following strategies: (a) searching the dbSNP database (http://www.ncbi.nlm.nih.gov/projects/SNP/), 1000 Genomes (http://www.1000genomes.org/) and finding SNPs with minor allele frequencies ≥0.02 among Han Chinese in Beijing and located within 2000 bp upstream and 300 bp downstream of the TGFBR2 and TGFBRAP1 genomic regions [18]; (b) under the experimental conditions for genotyping; and (c) a minor allele frequency (MAF) ≥0.05 and linkage disequilibrium (LD) *r*^2^ ≥ 0.8. Four TGFBRAP1 SNPs (rs17687727, rs75725426, rs2241797, and rs12476720) and eleven TGFBR2 SNPs (rs1835538, rs9881945, rs4522809, rs11924422, rs12493607, rs1808602, rs114342639, rs3773644, rs3773652, rs2043136, and rs876688) were examined in the current study ([Supplementary-material supplementary-material-1] Table).

### 2.5. Statistical Analysis

The demographic and clinical data of the enrolled patients in the ATDILI group and in the non-ATDILI group were compared using the chi-square test and *t*-test by SPSS version 17.0. The Hardy–Weinberg equilibrium (HWE) for all SNPs in the controls was assessed by Plink version 1.07. Associations between SNPs and ATDILI were evaluated using the unconditional logistic regression after adjusting for age and gender by Plink version 1.07. The odds ratio (OR) with 95% confidence interval (CI) was used as a measure of associations. The linkage disequilibrium (LD) and haplotype analysis were conducted by Haplotype version 4.2. Multifactor Dimensionality Reduction Software (version 3.0.2) was used to analyse the SNP-SNP interactions associated with ATDILI [19]. Two-sided values of *p* < 0.05 were considered statistically significant.

## 3. Results

### 3.1. Demographic and Clinical Characteristics of the Subjects

In total, 746 TB patients were included in this prospective study. The overall incidence rate of ATDILI was 15.82% (118/746) among the patients. There was no difference in age (*p*=0.285) or gender (*p*=0.801) between the patients with ATDILI and patients without ATDILI. Compared with patients without ATDILI, patients with ATDILI showed a tendency of higher percentage of fever and weight loss (*p*=0.016 and *p*=0.036) and a different proportion of tuberculosis subtype (*p* < 0.001). The ATDILI group also had a higher frequency of elevated serum levels of ALT (*p* < 0.001), AST (*p* < 0.001), ALP (*p* < 0.001), TBIL (*p*=0.002), IBIL (*p*=0.049), uric (*p*=0.008), and GGT (*p*=0.021). Among the ATDILI group, 70.34%, 17.80%, and 11.86% patients presented mild, moderate, and severe hepatotoxicity, respectively, without differences in age or gender; 39.83%, 16.10%, 17.80%, and 26.27% patients developed hepatocellular injury, cholestatic injury, mixed injury, and injury, respectively, with unknown classification. Demographic, clinical characteristics, laboratory indicators, severity, and clinical phenotype of patients are displayed in Table 1.

### 3.2. SNP Allele, Genotype, Genetic Model, and Haplotype Analysis

Genotyping of selected SNPs was successfully completed for all 118 patients in the ATDILI group and 628 patients in the non-ATDILI group. To ensure the repeatability and stability of genotyping, 30 samples were randomly selected for double-blind experiments, and all the genotype calling success rates were greater than 99.0%. None of the SNP genotype distributions deviated from the Hardy–Weinberg equilibrium (HWE), except rs2043136 (*p*=0.033). The distributions of genotype and allele frequencies of all fifteen SNPs are depicted in Table 2. For the rs17687727 locus, the proportions of the A allele were 46/234 (19.66%) in the ATDILI group and 171/1253 (13.73%) in the non-ATDILI group compared with the G allele (OR 1.55; 95% CI: 1.08–2.22, *p*=0.016). The occurrence of the AA genotype seemed more common in the ATDILI group (4/117, 3.42%) compared with the non-ATDILI group (11/627, 1.75%), but there was no significant difference (*p*=0.055). For other SNPs, no allele or genotype differences were found between the two groups (all *p* > 0.05).

Three genetic models were constructed to compare the significance of each SNP: dominant, recessive, and additive patterns. In line with the abovementioned findings, as shown in Table 3, rs17687727 in the dominant model (OR 1.634; 95% CI: 1.076–1.634; *p*=0.021) and additive model (OR 1.559; 95% CI: 1.083–2.246; *p*=0.017) showed statistical significance between the two groups. No genetic model was associated with ATDILI in other SNPs, even marginally.

We next constructed the haplotype to analyse whether there was additive association among selected SNPs. One haplotype consisted of rs2241797 and rs12476720 in TGFBRAP1, and three haplotypes consisting of rs11924422 and rs12493607, rs1808602 and rs114342639, and rs3773652 and rs2043136 in TGFBR2 (*D*′ > 0.80) were constructed with a frequency >0.05 and in a strong linkage disequilibrium state by calculating the pairwise *r*^2^ coefficient (*r*^2^ > 0.80). However, none was associated with ATDILI (*p* > 0.05).  Table 4 shows the association of the haplotypes of TGFBRAP1 and TGFBR2 with the risk of ATDILI. [Supplementary-material supplementary-material-1] Figure and [Supplementary-material supplementary-material-1] Figure depict the loci of TGFBRAP1 and TGFBR2 in the linkage disequilibrium block risk.

### 3.3. SNP-SNP Interactions with the Risk of ATDILI

We carried out a multifactor dimensionality reduction (MDR) analysis with all fifteen SNPs to investigate potential genetic interactions associated with ATDILI. We limited the interaction models from two-way to nine-way and linear regression for score calculation. However, we did not identify any multilocus model with receivable cross-validation consistency (from 3/10 to 6/10). Moreover, all these models did not reach the threshold value of statistical significance (all *p* > 0.05[Supplementary-material supplementary-material-1] Table).

### 3.4. The Relationship between Genetic Polymorphism and ATDILI Laboratory Test Indicators

Genetic polymorphism not only affects disease susceptibility but also has a certain correlation with the clinical features of the disease, which may affect different clinical characteristics of individuals. In this study, as shown in Table 5, the positive site rs17687727 in TGFBRAP1 and liver function-related laboratory test indicators indicated that the patients with the AA genotype had the highest AST 200.50 (100.50–276.50), whereas patients with the GA and GG genotypes had AST values of 83.00 (38.50–160.25) and 115.00 (72.50–217.00), respectively.

## 4. Discussion

The TGF-*β*1/smad signalling pathway can regulate liver homeostasis [9], although the distinct role of TGFBR2 and TGFBRAP1 in the TGF-*β*1/smad signalling pathway had been observed previously, playing a vital role in liver fibrosis and hepatocarcinogenesis [9]. No genetic association study was conducted to evaluate the correlation of TGFRB2 and TGFBRAP1 polymorphisms with ATDILI. In the present study, we first revealed that the A variants at rs17687727 loci were significantly associated with an increased risk for ATDILI in the Han Chinese population.

The TGFBRAP1 gene (Gene ID: 9392) maps to chromosome 2 at q12.1 and spans 80.29 kbp. No study on rs17687727 has been reported yet. A G > A mutation of rs17687727 located at the 3′ UTR of the TGFBRAP1 gene would influence the combined functions of the miRNAs. We searched the miRNA target gene prediction website database (http://www.targetscan.org) and found that TGFBRAP1 and miR-122 had potential binding sites ([Supplementary-material supplementary-material-1] Figure). MiR-122, which accounts for approximately 70% of the total miRNA in the adult liver, is involved in cell cycle progression, hepatocellular carcinogenesis, lipid metabolism, and fibrosis [20], so it was considered to have a high specificity in drug-induced liver injury with modest positive diagnostic effects [20, 21]. MiR-122 might inhibit hepatocellular carcinoma progression by downregulating TGFBRAP1 in the presence of the hepatitis C virus core, suggesting that the TGF-*β*/smad signalling pathway may be related to the expression level of miR-122, which plays an important role in drug-induced liver injury [22–24]. Exposure to TGF-*β* led to significant downregulation of miR-122. Furthermore, reintroduction of miR-122 suppressed TGF-*β*-induced expression of fibrosis-related genes in hepatic fibrogenesis [25]. Investigations have identified the ratio of miR-122/miR-155 as potential biomarkers for the early diagnosis of isoniazid-induced liver injury in mice [26]. In our study, we also found that the genetic polymorphism of TGFBRAP1 was related to the clinical features of liver injury and that patients with the AA genotype had a higher AST than patients with the GA and GG genotypes. Whether this regulation is also modified by miR-122 is worth exploring. Considering the haplotype is a combination of specific alleles at neighbouring genes that tend to be inherited together, multiple SNPs may “tag” an untyped variant more effectively than a single-typed variant. The subset of SNPs used in such an approach is called “haplotype tagging” SNPs [27]. We also generated a regional LD plot (http://www.internationalgenome.org) for rs17687727 to search for the “haplotype tagging” SNPs. Two estimated loci (rs34686799 and rs10176000) with high LD (*r*^2^ > 0.8) were found in the intron region, but no clear biological significance was found in these sites. In summary, taking the spatiotemporal orchestration of TGF-*β* signalling at different stages of liver injury, its cross-talk with several signalling pathways, and even its interplay with posttranslational modification into consideration [8], the role of the TGF-*β*/smad signalling pathway in ATDILI is obscure. Our study found that a variant of rs17687727 in the 3′ UTR region of the TGFBRAP1 gene was associated with susceptibility to ATDILI and suggested that fine mapping and further functional studies are necessary to evaluate the genetic effect of TGFBRAP1 and its potential regulatory mechanism on ATDILI.

The TGFBR2 gene (gene ID: 7048) is located on chromosome 3 at p24.1 and spans 87.65 kbp. Genotyping results showed that the rs4522809G allele was associated with ascending thoracic aorta with significantly higher TGF-*β*1 concentrations [28]. rs4522809 was found to have a strong predictive role in the regulation of osteopontin expression [29]. Associations of rs4522809 were meta-analysed with data from the NCI Polish Breast Cancer Study and published data from the Breast Cancer Association Consortium, which found a weak association [29]. For rs12493607, studies focused on the susceptibility to breast cancer with controversial results [30–32]. rs876688 has been researched in oral facial clefts, and no correlation was found [33]. We did not find any positive results for the SNPs in the TGFBR2 genetic region. One possible reason to explain this lack of association is that the TGF-*β*/smad signalling pathway involves different mechanisms in acute and chronic liver injuries. In brief, TGF-*β* plays a dual role in the control of proliferation and apoptosis. On the one hand, early on, it induces intracellular signals that mediate cell cycle arrest and apoptosis; on the other hand, at later times, it activates proliferative and antiapoptotic signals through activation of the EGFR pathway, especially as a central regulator in chronic liver disease contributing to fibrogenesis through inflammation [34]. As most of the ATDILI cases appeared within sixteen weeks (range: 6 weeks–6 months) after the start of the combined therapy, it is reasonable to speculate that it was mainly acute liver injury [3]. Therefore, TGFBR2 or its genetic variation may not play a pivotal role in this specific pathway. Second, TGF-*β* alone does not direct normal liver development. A hepatocyte growth factor (HGF) mediated smad-independent pathway is able to rescue the liver phenotype in SMAD2/3 mutants [35].

Given that combined analyses of SNPs may display a more complete picture of the candidate genes [27], we further conducted a haplotype analysis and a SNP-SNP interaction analysis of the selected tagSNPs. Neither a haplotype nor a joint effect was found in association with ATDILI, which explained on another level that the TGF-*β*/smad signalling pathway is related to ATDILI but may not be the main pathway.

There are several strengths of our study. First, our prospective study included patients from the West China Hospital, the largest medical centre in western China, which has surveillance of ATDILI with strict criteria to avoid misclassification and inclusion criteria. We excluded people with hepatitis B virus (HCV) or hepatitis C virus (HCV), as well as HIV coinfection, which were shown to be risk factors for ATDILI. Second, the laboratory for testing is one of the advanced and comprehensive laboratories integrating clinical, scientific research and teaching in China. The laboratory is also certified by the American Association of Pathologists (CAP). All the test data had good quality control and reliability. Third, people who were collecting and sorting clinical data and people who were responsible for laboratory data worked independently in this study to minimize potential bias. These differences may make the conclusions of our study more persuasive and representative to some degree.

There were several limitations in our study. First, we focused on ATDILI induced by first-line antituberculosis regimens and the genetic risk factors of TGFBR2 and TGFBRAP1 only, without assessment of other relevant genes, environmental risks, and comorbid conditions (malnutrition, alcoholism, chronic hepatitis C and chronic hepatitis B infection, HIV infection, and preexisting liver disease), as well as epigenetic modification. For example, association of genetic polymorphisms of the NAT2 gene with “slow acetylation” phenotypes has been clearly documented to increased risk of ATDILI [7]. It is an excellent discovery on the drug metabolism pathway. However, TGFBRAP1 and TGFBR2 may play a role in ATDILI by the TGF-*β*/smad signalling pathway through potential immune regulation. In our study, we did not analyse the gene polymorphisms of NAT2 gene simultaneously, so we did not analyse the relationship between rs17687727 and slow acetylator status, and the correlation between the TGF-*β*/smad signalling pathway and isoniazid acetylation is still poorly understood. Concomitant viral hepatitis infection may be another confounding factor in ATDILI, and the risk of ATDILI is directly related to the viral load [3]. It is difficult to perform real-time fluorescent PCR testing for every patient to detect the precise level of HBV-DNA/HCV-RNA concentration. Meanwhile, the ALT, AST, and ALP levels of patients with viral hepatitis also have an increased likelihood, which makes it more difficult to do causal judgement of liver damage caused by antituberculosis drugs or hepatitis. To avoid bias and confounding variables caused by different viral loads and/or hepatitis progression itself, we excluded patients with hepatitis B virus or hepatitis C virus in the study. Second, all the samples in our study were Han Chinese in western China and not large enough to detect a rare risk allele in other ethnicities. No differences in age and gender were found between the ATDILI group and the control group in our population. Older age is associated with decreased liver blood flow and changes in the drug distribution and metabolism, thus potentially reducing the effective clearance of the drugs [3]. To make this point clear, we further analysed the age composition according to the severity of liver injuries, and no significant differences were observed (Table 1). To explore the correlation between age and ATDILI in the Chinese population, we looked for genetic polymorphism studies of ATDILI based on the Chinese Han population. Although the target genes of the study are different, these studies did not have a significant difference in gender or age, neither [36–39]. Taken together, we hypothesized that due to the genetic backgrounds of different ethnic groups, perhaps the correlation between age and ATDILI for the Chinese Han population is not as obvious as other ethnic groups. However, it cannot be ruled out that the undetected correlation between age and ATDILI is due to the limited sample size. Furthermore, extended validation in multicentre and enlarged sample studies in other cohorts is needed to identify the association between target and ATDILI, plus functional verification test in vitro and vivo.

In conclusion, we found that genetic polymorphisms of rs17687727 in the TGFBRAP1 gene influenced the susceptibility to ATDILI in first-line antituberculosis combination treatment in a Chinese population. We believe that mapping the TGFBRAP1 variants in a larger population along with functional verifications will further explore the important role of the TGF-*β*1/smad signalling pathway in the process. These findings provide novel insight into better understanding the molecular mechanisms of ATDILI and shed light on still unrecognized candidate targets for developing better personalized therapy and successful treatment in ATDILI.

## Figures and Tables

**Table 1:** Demographic characteristics, clinical characteristics, and laboratory indicators of enrolled patients.

Group	Non-ATDILI (*n* = 628)		ATDILI (*n* = 118)				*p* value	
*General data*								
Age (years)^a^	40.92 ± 15.72		42.85 ± 18.44				0.284	
Gender (male/female)^c^	375 (59.71%)	253 (40.28%)	69 (58.47%)		49 (41.52%)		0.801	
Smoking (no/yes)^c^	407 (64.80%)	221 (35.19%)	80 (67.79%)		38 (32.20%)		0.532	
Drinking (no/yes)^c^	465 (74.04%)	163 (25.95%)	83 (70.33%)		35 (29.66%)		0.464	

*Tuberculosis subtype, n (%)*								
PTB^c^	520	82.80%	79		66.95%		<**0.001**	
EPTB^c^	43	6.85%	15		12.71%			
PTB and EPTB^c^	65	10.35%	24		20.34%			
General symptoms (no/yes)^c^	135 (19.62%)	492 (80.37%)	23 (19.49%)		95 (80.51%)		0.567	
Fever (no/yes)^c^	344 (54.78%)	284 (45.22%)	50 (42.37%)		68 (57.62%)		**0.016**	
Weight loss (no/yes)^c^	367 (58.43%)	261 (41.56%)	82 (69.49%)		36 (30.50%)		**0.036**	
Night sweat (no/yes)^c^	433 (68.94%)	195 (31.05%)	86 (72.88%)		32 (21.12%)		0.446	
Fatigue (no/yes)^c^	462 (73.57%)	166 (26.43%)	85 (72.03%)		33 (27.97%)		0.716	
Poor appetite (no/yes)^c^	374 (59.55%)	254 (40.45%)	69 (58.47%)		49 (41.52%)		0.859	
Local infection (no/yes)^c^	134 (21.34%)	494 (78.66%)	24 (20.34)		94 (79.66%)		0.758	

*Laboratory examinations*	Mean ± SD or *p*50 (*p*25–*p*75)							
RBC (×1012/L)^a^	4.28 ± 0.68		4.31 ± 0.74				0.481	
HB (g/L)^a^	122.06 ± 20.58		122.87 ± 22.11				0.717	
HCT (L/L)^a^	0.36 ± 0.06		0.38 ± 0.06				0.069	
PLT (×10^9^/L)^b^	232.50 (172.75–297.25)		236.50 (184.00–321.75)				0.134	
WBC (×10^9^/L)^b^	6.51 (5.17–8.44)		6.57 (4.99–7.96)				0.761	
Neutrophils (×10^9^/L)^a^	5.10 ± 2.73		5.23 ± 2.89				0.631	
Monocytes (×10^9^/L)^a^	1.26 ± 0.62		1.29 ± 0.79				0.625	
Lymphocytes (×10^9^/L)^a^	0.50 ± 0.25		0.55 ± 0.29				0.099	
Neutrophils (%)^a^	70.13 ± 11.54		70.49 ± 11.50				0.760	
Monocytes (%)^a^	7.30 ± 2.37		7.74 ± 2.62				0.077	
Lymphocytes (%)^b^	17.5 (12.18–25.68)		16.25 (12.58–25.58)				0.527	
TBIL (*μ*mol/L)^b^	8.70 (6.30–12.10)		10.05 (7.50–14.13)				0.002	
DBIL (*μ*mol/L)^b^	3.45 (2.50–5.40)		3.55 (2.38–5.60)				0.126	
IBIL (*μ*mol/L)^b^	4.80 (3.40–7.03)		5.70 (3.98–7.95)				**0.049**	
ALT (IU/L)^b^	15.00 (10.00–21.00)		28.00 (15.75–38.00)				<**0.001**	
AST (IU/L)^b^	19.50 (16.00–25.00)		27.00 (20.00–34.00)				<**0.001**	
TP (g/L)^a^	68.82 ± 9.15		69.42 ± 8.42				0.508	
ALB (g/L)^a^	37.89 ± 6.90		38.64 ± 7.35				0.248	
GLB (g/L)^a^	30.93 ± 7.02		30.78 ± 6.65				0.829	
GLU (mmol/L)^b^	5.14 (4.71–5.89)		5.15 (4.64–5.95)				0.41	
UREA (mmol/L)^b^	4.05 (3.15–5.30)		3.92 (2.90–5.24)				0.299	
CREA (*μ*mol/L)^b^	60.45 (49.00–73.20)		57.50 (47.78–67.00)				0.601	
CYS-C (mg/L)^b^	0.92 (0.79–1.06)		0.91 (0.81–1.04)				0.975	
Uric (*μ*mol/L)^a^	331.51 ± 155.30		291.29 ± 125.98				**0.008**	
TG (mmol/L)^b^	1.06 (0.80–1.43)		0.99 (0.81–1.31)				0.469	
CHOL (mmol/L)^a^	3.96 ± 1.058		3.96 ± 1.206				0.966	
HDL-C (mmol/L)^a^	1.08 (0.82–1.41)		1.12 (0.85–1.48)				0.811	
LDL-C (mmol/L)^b^	2.21 (1.69–2.77)		2.20 (1.79–2.72)				0.575	
ALP (IU/L)^b^	79.00 (64.00–98.00)		85.50 (68.50–106.00)				**0.021**	
GGT (IU/L)^b^	29.00 (19.00–48.00)		42.50 (26.00–78.00)				<**0.001**	
CRP (mg/L)^b^	12.25 (2.67–37.43)		9.74 (2.30–39.23)				0.961	
ESR (mm/h)^b^	33.50 (14.75–64.00)		38.50 (20.50–63.00)				0.173	

*Severity*			*N*	Age (years)	*p*	Gender (*N*)		*p*
						Male	Female	
Mild			83	40.42 ± 16.48	0.888	53	30	0.117
Moderate			21	42.19 ± 14.04		11	10	
Severe			14	41.57 ± 14.78		5	9	

TB, tuberculosis; PTB, pulmonary tuberculosis; EPTB, extrapulmonary tuberculosis. ^a^Data are shown as mean ± standard deviation; ^b^data are shown as median (interquartile range); ^c^data are shown as number of cases (frequency).

**Table 2:** The distributions of allele and genotype frequencies of all fifteen SNPs.

Gene	dbSNP		Allele			*p*	*p* ^HWE^	Genotype		*p*
		Allele	ATDILI (*n*, %)	Non-ATDILI (*n*, %)	OR (95% CI)			ATDILI	Non-ATDIH	
			1/2	1/2				11/12/22	11/12/22	
TGFBRAP1	rs17687727	G > A	46/188	171/1083	1.55 (1.08–2.22)	**0.016**	0.883	4/38/75	11/149/467	0.055
	rs75725426	A > G	27/205	140/1116	1.05 (0.67–1.62)	0.827	0.096	2/23/91	12/116/500	0.936
	rs2241797	T > C	64/170	340/912	1.01 (0.73–1.38)	0.951	0.578	8/48/61	50/240/336	0.821
	rs12476720	A > G	113/123	622/634	0.93 (0.70–1.23)	0.643	1.000	28/57/33	153/316/159	0.832

TGFBR2	rs1835538	G > A	29/207	188/1068	0.79 (0.52–1.20)	0.283	0.638	2/25/91	12/164/452	0.512
	rs9881945	G > T	36/200	180/1074	1.07 (0.72–1.58)	0.718	0.744	3/30/85	14/152/461	0.937
	rs4522809	A > G	69/167	382/868	0.93 (0.69–1.27)	0.685	0.924	6/57/55	59/264/302	0.216
	rs11924422	C > A	69/167	383/869	0.93 (0.69–1.27)	0.678	0.258	9/51/58	52/279/295	0.912
	rs12493607	C > G	78/158	412/838	1.00 (0.74–1.35)	0.978	0.651	11/56/51	65/282/278	0.874
	rs1808602	A > G	102/134	575/677	0.89 (0.67–1.18)	0.443	0.809	19/64/35	130/315/181	0.497
	rs114342639	G > T	46/190	260/994	0.92 (0.65–1.31)	0.664	0.903	4/38/76	26/208/393	0.897
	rs3773644	C > T	88/148	500/754	0.89 (0.67–1.19)	0.456	0.454	18/52/48	95/310/222	0.508
	rs3773652	G > A	123/113	602/648	1.17 (0.88–1.54)	0.264	0.575	32/59/27	141/320/164	0.509
	rs2043136	A > G	17/101	102/524	0.94 (0.71–1.25)	0.680	0.033	17/64/37	102/334/190	0.874
	rs876688	G > A	66/170	297/959	1.25 (0.91–1.71)	0.155	0.438	11/44/63	31/235/362	0.156

*p*: *p* value was calculated using logistic regression analysis. *p*^HWE^: *p* value of Hardy–Weinberg equilibrium. HWE was assessed by the *χ*^2^ goodness-of-fit test based on the genotype distributions in this study. The significance of bold in the table means *p* value < 0.05. “1” designates the mutant allele and “2” designates the wild allele; 11 = mutant homozygote; 12 = heterozygote; 22 = wild homozygote.

**Table 3:** Genetic models of related SNPs associated with ATDILI in tuberculosis patients.

Gene	dbSNP	Dominant model		Recessive model		Additive model	
		OR (95% CI)	*p*	OR (95% CI)	*p*	OR (95% CI)	*p*
TGFBRAP1	rs17687727	1.634 (1.076–1.634)	**0.021**	1.982 (0.620–6.335)	0.248	1.559 (1.083–2.246)	**0.017**
	rs75725426	1.073 (0.661–1.073)	0.774	0.900 (0.198–4.078)	0.891	1.047 (0.684–1.601)	0.832
	rs2241797	1.064 (0.716–1.064)	0.759	0.845 (0.390–1.833)	0.670	1.010 (0.740–1.377)	0.951
	rs2576736	0.793 (0.526–0.793)	0.268	0.908 (0.397–2.076)	0.819	0.847 (0.607–1.182)	0.329
	rs12476720	0.873 (0.562–0.873)	0.546	0.965 (0.608–1.532)	0.882	0.936 (0.708–1.237)	0.643
	rs2679876	0.816 (0.537–0.816)	0.342	0.632 (0.219–1.820)	0.395	0.820 (0.576–1.168)	0.272

TGFBR2	rs1835538	0.762 (0.479–1.211)	0.250	0.885 (0.195–4.007)	0.874	0.792 (0.519–1.209)	0.280
	rs9881945	1.078 (0.694–1.673)	0.737	1.142 (0.323–4.038)	0.836	1.073 (0.729–1.578)	0.720
	rs4522809	1.071 (0.722–1.589)	0.733	0.513 (0.216–1.219)	0.131	0.937 (0.688–1.276)	0.682
	rs11924422	0.922 (0.622–1.367)	0.685	0.911 (0.436–1.904)	0.805	0.934 (0.683–1.278)	0.670
	rs12493607	1.052 (0.707–1.565)	0.800	0.885 (0.452–1.734)	0.723	1.004 (0.743–1.356)	0.978
	rs1808602	0.964 (0.626–1.484)	0.869	0.732 (0.432–1.241)	0.246	0.893 (0.672–1.188)	0.437
	rs1078985	0.959 (0.619–1.487)	0.854	1.583 (0.572–4.376)	0.376	1.025 (0.706–1.488)	0.896
	rs9847368	0.864 (0.535–1.394)	0.549	1.066 (0.230–4.926)	0.935	0.891 (0.578–1.375)	0.604
	rs114342639	0.929 (0.615–1.399)	0.721	0.811 (0.277–2.368)	0.701	0.924 (0.650–1.315)	0.663
	rs3773644	0.799 (0.534–1.195)	0.275	1.008 (0.583–1.742)	0.977	0.894 (0.669–1.196)	0.452
	rs3773652	1.199 (0.753–1.909)	0.444	1.277 (0.816–1.997)	0.283	1.176 (0.887–1.558)	0.260
	rs2043136	0.954 (0.624–1.459)	0.827	0.864 (0.496–1.508)	0.608	0.936 (0.696–1.26)	0.665
	rs876688	1.188 (0.800–1.763)	0.392	1.98 (0.965–4.059)	0.062	1.259 (0.917–1.727)	0.153

*p*: *p* value was calculated using logistic regression analysis.

**Table 4:** Analysis of haplotypes with the risk of ATDILI.

Gene	SNP	Haplotype^*∗*^	Frequency	*p*
TGFBRAP1	rs2241797 : rs12476720	GA	0.497	0.575
		AG	0.261	0.963
		GG	0.231	0.457

TGFBR2	rs11924422 : rs12493607	AG	0.649	0.804
		CC	0.281	0.922
		AC	0.048	0.734
	rs1808602 : rs114342639	AC	0.536	0.485
		GC	0.259	0.682
		GA	0.196	0.618
	rs3773652 : rs2043136	AA	0.483	0.332
		GG	0.423	0.573
		GA	0.089	0.325

^*∗*^Ratio is shown by CC frequencies.

**Table 5:** Quantitative indicator comparisons among genotypes of rs17687727 in TGFBRAP1.

Laboratory indicators	Genotype			*p*
	GG	GA	AA	
TBIL (*μ*mol/L)^a^	12.15 (7.15–18.44)	13.40 (9.70–19.55)	10.35 (6.85–17.67)	0.48
DBIL (*μ*mol/L)^a^	5.65 (3.25–10.40)	5.60 (3.95–8.79)	3.90 (3.80–4.98)	0.45
IBIL (*μ*mol/L)^a^	4.90 (3.57–8.12)	6.60 (4.74–10.2)	6.45 (3.00–12.75)	0.08
ALT (IU/L)^a^	108.00 (50.50–191.75)	164.00 (105.00–316.00)	91.00 (38.00–250.50)	0.03
AST (IU/L)^a^	83.00 (38.50–160.25)	115.00 (72.50–217.00)	200.50 (100.50–276.50)	0.03
ALP (IU/L)^a^	108.5 (75.25–189.25)	98.00 (71.50–126.50)	97.50 (77.75–129.25)	0.32

^a^Data are shown as median (interquartile range).

## Data Availability

The data used to support the findings of this study are included with in the article and the supplementary information file.

## Supplementary Materials

Supplementary Materials S1 Table: Candidate single nucleotide polymorphism of TGFBRAP1 and TGFBR2. The location (GRCh38.p7) and region of the SNPs, and the MAF of the SNPs in 1000 Genomes (East Asia) and in our study. S2 Table: SNP-SNP interactions analysed with Multifactor Dimensionality Reduction Software (version 3.0.2). S1 Figure: Flow diagram of the enrolment of the study population. S2 Figure: Haplotype analysis for the candidate SNPs of TGFBRAP1 based on linkage disequilibrium (LD) plots. S3 Figure: Haplotype analysis for the candidate SNPs of TGFBR2 based on linkage disequilibrium (LD) plots.
