# Supplementary material for: The Variant at TGFBRAP1 but Not TGFBR2 Is Associated with Antituberculosis Drug-Induced Liver Injury
Source: Evid Based Complement Alternat Med. 2019 Aug 22;2019:1685128. doi: 10.1155/2019/1685128 (PMC6724436; doi:10.1155/2019/1685128)
Supplement: Supplementary Materials — S1 Table: Candidate single nucleotide polymorphism of TGFBRAP1 and TGFBR2. The location (GRCh38.p7) and region of the SNPs, and the MAF of the SNPs in 1000 Genomes (East Asia) and in our study. S2 Table: SNP-SNP interactions analysed with Multifactor Dimensionality Reduction Software (version 3.0.2). S1 Figure: Flow diagram of the enrolment of the study population. S2 Figure: Haplotype analysis for the candidate SNPs of TGFBRAP1 based on linkage disequilibrium (LD) plots. S3 Figure: Haplotype analysis for the candidate SNPs of TGFBR2 based on linkage disequilibrium (LD) plots. [file 1685128.f1.zip › 1685128.f1/S1 table Candidate single nucleotide polymorphism of TGFBRAP1 and TGFBR2.docx]

| Supplementary Table 1.Candidate single nucleotide polymorphism of TGFBRAP1 and TGFBR2. | | | | | |  |
| --- | --- | --- | --- | --- | --- | --- |
| gene | dbSNP | allele | Location(GRCh38.p7) | region | MAF | MAF* |
| TGFBRAP1 | rs17687727 | G>A | chr2:105249510 | 3 Prime UTR Variant | A=0.191 | 0.146 |
|  | rs75725426 | A>G | chr2:105265029 | Non Coding Transcript Variant | G=0.090 | 0.112 |
|  | rs2241797 | T>C | chr2:105269504 | Missense Variant | C=0.276 | 0.271 |
|  | rs12476720 | A>G | chr2:105298671 | Synonymous Variant | G=0.430 | 0.493 |
| TGFBR2 | rs1835538 | G>A | chr3:30613989 | Intron Variant | \|  \| A=0.141 \| \| --- \| --- \| | 0.145 |
|  | rs9881945 | G>T | chr3:30620957 | Intron Variant | T=0.152 | 0.145 |
|  | rs4522809 | A>G | chr3:30627192 | Intron Variant | G=0.290 | 0.303 |
|  | rs11924422 | C>A | chr3:30635992 | Intron Variant | C=0.283 | 0.303 |
|  | rs12493607 | C>G | chr3:30641447 | Intron Variant | G=0.294 | 0.329 |
|  | rs1808602 | A>G | chr3:30648932 | Intron Variant | G=0.469 | 0.455 |
|  | rs114342639 | G>T | chr3:30666623 | Intron Variant | T=0.170 | 0.205 |
|  | rs3773644 | C>T | chr3:30670852 | Intron Variant | T=0.418 | 0.394 |
|  | rs3773652 | G>A | chr3:30677450 | Intron Variant | A=0.458 | 0.487 |
|  | rs2043136 | A>G | chr3:30678812 | Intron Variant | G=0.453 | 0.427 |
|  | rs876688 | G>A | chr3:30684284 | Intron Variant | A=0.226 | 0.243 |
| MAF: minor allele frequency in 1000 Genomes (East Asia) <https://www.ncbi.nlm.nih.gov/snp>  *:MAF Calculated by Haploview software in our study | | | | | | |
