# Supplementary material for: The Variant at TGFBRAP1 but Not TGFBR2 Is Associated with Antituberculosis Drug-Induced Liver Injury
Source: Evid Based Complement Alternat Med. 2019 Aug 22;2019:1685128. doi: 10.1155/2019/1685128 (PMC6724436; doi:10.1155/2019/1685128)
Supplement: Supplementary Materials — S1 Table: Candidate single nucleotide polymorphism of TGFBRAP1 and TGFBR2. The location (GRCh38.p7) and region of the SNPs, and the MAF of the SNPs in 1000 Genomes (East Asia) and in our study. S2 Table: SNP-SNP interactions analysed with Multifactor Dimensionality Reduction Software (version 3.0.2). S1 Figure: Flow diagram of the enrolment of the study population. S2 Figure: Haplotype analysis for the candidate SNPs of TGFBRAP1 based on linkage disequilibrium (LD) plots. S3 Figure: Haplotype analysis for the candidate SNPs of TGFBR2 based on linkage disequilibrium (LD) plots. [file 1685128.f1.zip › 1685128.f1/S2 table The SNP-SNP interactions.docx]

| S2 Table: SNP-SNP interactions analyzed with MDR |  |  |  |
| --- | --- | --- | --- |
| Best models | CVC | TBA | P value^*^ |
| rs11924422 rs3773652 | 5/10 | 0.497 | 0.623 |
| rs11924422 rs114342639 rs3773652 | 5/10 | 0.490 | 0.623 |
| rs11924422 rs1808602 rs3773644 rs3773652 | 3/10 | 0.472 | 0.989 |
| rs4522809 rs11924422 rs1808602 rs3773652 rs2043136 | 3/10 | 0.478 | 0.828 |
| rs4522809 rs11924422 rs1808602 rs3773644 rs3773652 rs2043136 | 4/10 | 0.506 | 0.623 |
| rs4522809 rs11924422 rs1808602 rs3773644 rs3773652 rs2043136 rs17687727 | 6/10 | 0.530 | 0.172 |
| rs4522809 rs11924422 rs1808602 rs3773644 rs3773652 rs2043136 rs17687727 rs12476720 | 4/10 | 0.502 | 0.828 |
| rs4522809 rs11924422 rs1808602 rs3773644 rs3773652 rs2043136 rs17687727 rs2241797 rs12476720 | 4/10 | 0.507 | 0.828 |
| CVC: Cross Validation Consistency, TBA: Testing Balanced Accuracy. |  |  |  |
| * P-value based on 1000-fold permutation test. |  |  |  |
