# Supplementary material for: The Variant at TGFBRAP1 but Not TGFBR2 Is Associated with Antituberculosis Drug-Induced Liver Injury
Source: Evid Based Complement Alternat Med. 2019 Aug 22;2019:1685128. doi: 10.1155/2019/1685128 (PMC6724436; doi:10.1155/2019/1685128)
Supplement: Supplementary Materials — S1 Table: Candidate single nucleotide polymorphism of TGFBRAP1 and TGFBR2. The location (GRCh38.p7) and region of the SNPs, and the MAF of the SNPs in 1000 Genomes (East Asia) and in our study. S2 Table: SNP-SNP interactions analysed with Multifactor Dimensionality Reduction Software (version 3.0.2). S1 Figure: Flow diagram of the enrolment of the study population. S2 Figure: Haplotype analysis for the candidate SNPs of TGFBRAP1 based on linkage disequilibrium (LD) plots. S3 Figure: Haplotype analysis for the candidate SNPs of TGFBR2 based on linkage disequilibrium (LD) plots. [file 1685128.f1.zip › 1685128.f1/S1 -S3 Figure.doc]

S1 Figure. Flow diagram of enrollment of the study population.

**Analysis**

**Enrollment**

Highly suspicious TB patients were consecutively enrolled from December 2016 to April 2018(n=1060)

Excluded (n=287 )

♦  without evidenced of tuberculosis (n= 243 )

♦  with HIV,HBV or HCV (n=16 )

♦  with abnormal liver function before treatment (n=28 )

Tuberculosis patients with anti-tuberculosis induced liver injury (n=118 )

Excluded (n=27 )

♦  lost follow up (n= 13 )

♦  with use of other hepatotoxic medicine (n=16 )

♦  use second-line anti-tuberculosis drugs (n=28 )

Receive standard anti-tuberculosis drugs

With good treatment Compliance

(n=746 )

Included with definite diagosis of tuberculosis

Six months follow-up treatment and data recording (n= 773 )

Tuberculosis patients without anti-tuberculosis induced liver injury (n=628 )

S2.Figure Haplotype analysis for the candidate SNPs of TGFBRAP1 based on Linkage disequilibrium (LD) plots .The strength of the LD between SNPs is indicated by the color scheme, measured using D’ and r^2^ statistics.

S3.Figure Haplotype analysis for the candidate SNPs of TGFBR2 based on Linkage disequilibrium (LD) plots .The strength of the LD between SNPs is indicated by the color scheme, measured using D’ and r^2^ statistics.
